# Squamous Cell Carcinoma of the Pancreas in a Patient with Germline *BRCA2* Mutation-Response to Neoadjuvant Radiochemotherapy

**DOI:** 10.1155/2014/860532

**Published:** 2014-05-19

**Authors:** Anne M. Schultheis, Gia Phuong Nguyen, Monika Ortmann, Wolfgang Kruis, Reinhard Büttner, Hans-Ulrich Schildhaus, Birgid Markiefka

**Affiliations:** ^1^Institute of Pathology, University Hospital Cologne, Kerpener Straße 62, 50924 Cologne, Germany; ^2^Evangelisches Krankenhaus Köln-Kalk, Cologne, Germany; ^3^Institute of Pathology, University Hospital Göttingen, Göttingen, Germany

## Abstract

Primary squamous cell carcinoma of the pancreas is a rare malignant neoplasia, accounting for approximately 0.5–2% of all malignant pancreatic tumors. These lesions are characterized by poor prognosis. Here we report on a case of a 57-year-old female patient with known *BRCA2* germline mutation presenting with primary squamous cell carcinoma of the pancreas as the only malignancy. The tumor was locally advanced at the first presentation but responded almost completely to neoadjuvant radio-chemotherapy. Our case highlights the facts (i) that pancreatic carcinomas belong to the tumor spectrum of patients with the *BRCA2*-associated hereditary breast and ovarian cancer syndrome (HBOC) and (ii) that tumors of the pancreas can represent the first or even the only manifestation of HBOC. Furthermore, this case of a nonkeratinizing squamous cell carcinoma indicates that HBOC-associated carcinomas of the pancreas might be characterized by a broader morphological spectrum than was previously thought. Since *BRCA* mutations cause deficiency of DNA double-strand breakage repair in tumors, neoadjuvant treatment regimens might become a reasonable option in HBOC-associated pancreatic carcinomas. To our knowledge, this is the first reported case of a primary pancreatic squamous cell carcinoma in a patient with this particular genetic background of *BRCA2*-associated HBOC.

## 1. Introduction

*BRCA2* is a tumor suppressor gene. Its germline mutations cause a tumor predisposition referred to as hereditary breast and ovarian cancer syndrome (HBOC, OMIM # 612555) [1]. Carriers of the mutated gene have a significantly increased risk of developing breast and ovarian cancers and further of tumors originating also from other organs, for example, stomach, larynx, prostate, and fallopian tube. Notably, pancreatic carcinomas are as well part of the HBOC syndrome. The estimated risk of developing pancreatic carcinomas is increased 3.5–10-fold in carriers of* BRCA2* mutations [1]. So far, all described* BRCA2* mutation-associated tumors of the pancreas showed the classical histomorphology of common ductal adenocarcinomas.

Primary squamous cell carcinoma of the pancreas is a rare malignancy, accounting for approximately 0.5–2% of all neoplasms of the exocrine pancreas with only 40 reported cases in the literature to date. These rare tumors are clinically characterized by poor prognosis [2]. Until present, squamous cell carcinomas of the pancreas have never been described in the context of germ line* BRCA2* mutations or within the tumor spectrum of the HBOC syndrome.

## 2. Case Presentation

### 2.1. Patient's History and Histologic Findings

**Figure 1:**
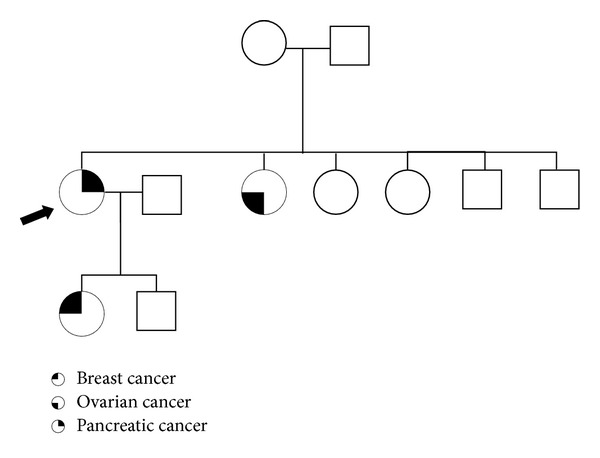
Pedigree of the affected family. Pedigree shows the three family members tested for* BRCA2* mutation and known tumor burden; arrow points to the index patient described in this report. Other family members were not tested or no information could be obtained. Notably, the three affected family members suffered from completely different tumor types which are, however, all parts of the HBOC tumor syndrome.

**Figure 2:**
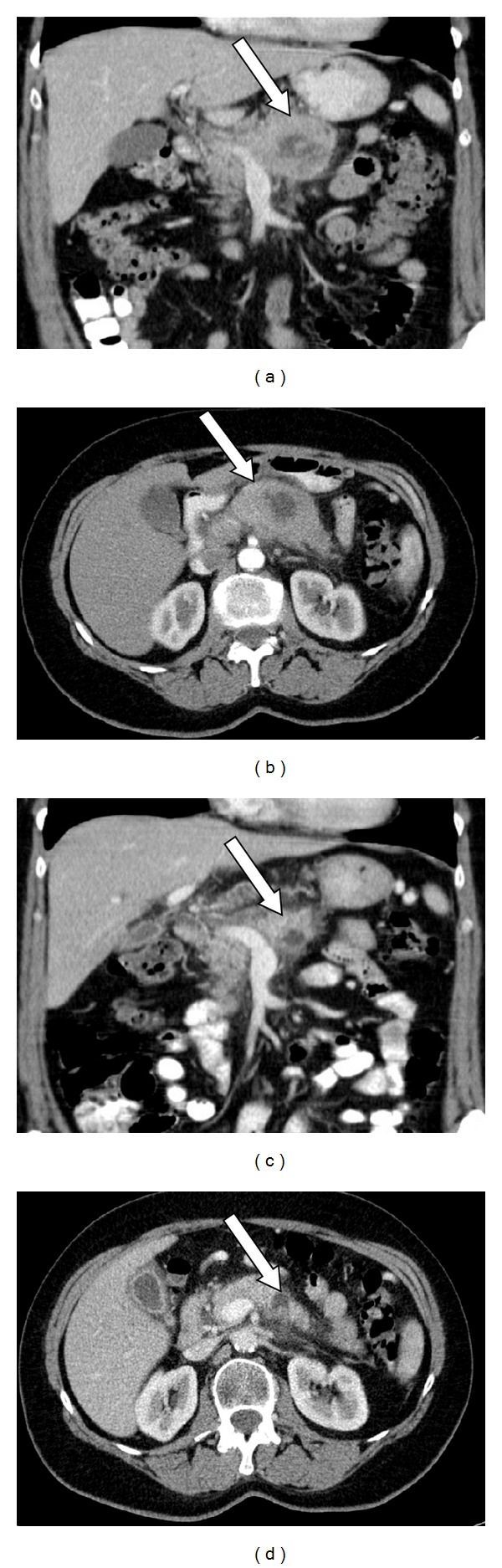
CT scans of the index patient. (a) (transversal image) and (b) (horizontal image) show the tumor's pretreatment extension with centrally cystic areas and signs of splenic vein infiltration; (c) (transversal image) and (d) (horizontal image) show tumor shrinkage after neoadjuvant radiochemotherapy; arrow is directed to tumor mass.

**Figure 3:**
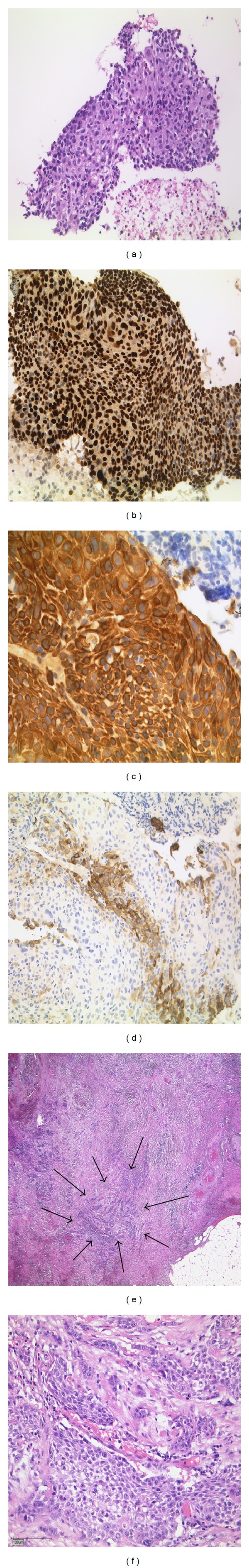
Histology and immunohistochemistry. Tumor biopsy (pretreatment): (a) solid mass of cohesive, discretely pleomorphic cells of intermediate size with hyperchromatic, pleomorphic nuclei (H&E, original magnification: ×100). (b) Immunohistochemistry revealed nuclear positivity for p63 (×200). Strong cytoplasmatic positivity for CK5/6, ((c), ×400). CK7 staining results in only focal weak positivity of few tumor cells ((d), ×200). Histology of resection specimen (after treatment). (e) Overview of tumor extension, large areas of fibrosis were seen; small circumscribed area of vital tumor is indicated by arrows (H&E, ×25). Closer view on vital tumor cells resembling pretreatment morphology ((f), H&E ×200).

A 57-year-old female patient consulted her physician for postprandial epigastric pain persisting over the last five weeks. No other physical complaints were reported. Her medical history revealed an incidental finding of a carcinoid tumor of the appendix as well as a known* BRCA2* mutation in exon 15 of the gene (nonsense mutation c.C7786T, p.R2520X). She had undergone mutational analysis because her sister had suffered from ovarian cancer at the age of 51 and her daughter from unilateral breast cancer at the age of 35. No other family members were tested or were known to have developed carcinomas (Figure 1). The index patient did not suffer from breast or ovarian cancer. At clinical examination the patient presented in good physical condition. The hematology exams revealed mild anemia with a hemoglobin value of 11.7 g/dL and a moderately increased CRP value of 21 mg/L. CA19.9 serum levels were normal. Ultrasound examination of the gastrointestinal tract revealed a pancreatic mass of 5-6 cm in diameter, located in the body and tail of the pancreas. CT scanning showed a solid tumor with central cystic structures and signs of infiltration of the splenic vein (Figure 2) as well as enlarged peripancreatic lymph nodes suspicious of locoregional metastases. Endosonographic biopsy was undertaken. Histologically, the tumor presented as a solid mass of cohesive, discretely pleomorphic cells of intermediate size with hyperchromatic, pleomorphic nuclei, with some containing a prominent nucleolus. The cytoplasm was eosinophilic and granular (Figure 3).

Based on extended disease, missing signs of distant metastases, and local inoperability of the tumor (clinical classification: cT3N1M0-stage IIB), primary neoadjuvant radiochemotherapy was recommended under respect of the patient's good physical condition and reports of good response rates in* BRCA2* mutation-associated carcinomas. A total of 54 Gy radiation dose plus gemcitabine 1/week 300 mg/m^2^ body surface was administered.

### 2.2. Immunohistochemistry and k-ras Mutational Analysis

Immunhistochemically, the tumor cells showed strong and homogenous positivity for CKAE1/3 (clone: AE1/3, DAKO, 1 : 200), CK5/6 (D5&16B4, CellMarque, 1 : 50), and p63 (A4A, Zeta Corp., 1 : 100). Focal positivity was found for CEA (II-7, DAKO, 1 : 200) and CA19.9 (1116-NS-19-9, DAKO, 1 : 800). Only few lesional cells were weakly immunopositive for CK7 (OV-TL, DAKO, 1 : 3200). According to morphology and immunohistochemical findings the tumor was classified as nonkeratinizing squamous cell carcinoma (Figure 3).

The molecular analysis of the tumor showed no k-ras mutation.

### 2.3. Patient's Follow-Up

After three months of neoadjuvant therapy, the tumor size was reduced to a radiographically detectable mass of 2.5 cm (Figure 2) and then was removed surgically. Histological examination of the complete resection specimen revealed more than 95% dense fibrosis in the previous tumor site with only small areas of vital tumor cells and no metastatic lymph nodes. Histomorphologically and immunhistochemically, the remaining tumor cells showed the same characteristics as initially described for the biopsy specimen (Figure 3). Pathological TNM classification was as follows: ypT1, ypN0 (0/12), R0, L0, and V0. The patient recovered from surgery and was stable for 5 months postoperatively. However, she was suffering from dysphagia and was unable to nourish herself, due to a radiation-associated stenosis of the duodenum. She suffered from weight loss which could be stabilized under supportive care and the symptoms were manageable for another 2 months. Seven months after surgery she developed a thrombosis of the subclavian vein, being treated with low dose heparine under which she developed a hematothorax. In spite of drainage and stopping of the bleeding, the patient could not be stabilized hemodynamically and died. Seven months after surgery, at time of death, there was still no evidence of tumor disease.

## 3. Discussion

*BRCA1 *and* BRCA2* are tumor suppressor genes encoding proteins involved in a common pathway of DNA double-strand repair. This particular DNA repair mechanism, also known as homologous recombination, uses the undamaged sister chromosome to repair mainly replication-associated DNA double-strand breaks [3]. The coding regions of* BRCA1* and* BRCA2* as well as the encoded proteins show no homology to each other, and the link between the two proteins is not well understood until now [3]. However, mutations in both genes share a common clinical disease phenotype, the so-called hereditary breast and ovarian cancer syndrome (HBOC), which is inherited in an autosomal dominant manner [3]. Carriers of the mutated gene show an increased risk of developing breast, ovarian, stomach, laryngeal, prostate, and pancreatic cancers, as well as cancer of the fallopian tube. HBOC syndrome accounts for 5–7% of all cases of breast cancer, and patients carrying the mutation have a lifetime risk for developing breast cancer of 50–80% and 30–50% for ovarian cancer (both* BRCA1* and* BRCA2*) [3]. Interestingly, for* BRCA1* mutation carriers, certain morphological subtypes of breast cancer, most frequently medullary carcinoma, as well as higher grade tumors, were reported to occur more commonly than in the sporadic counterparts. Furthermore,* BRCA1* mutation carriers seem to predominantly develop estrogen and progesterone receptor-negative and HER2-negative (triple-negative) breast cancer. In contrast,* BRCA2* mutation-associated breast tumors are not described to favor the development of any particular histopathological subtype of breast cancer [4–6].

In general, the frequency of developing malignancies other than breast and ovarian cancers differs between* BRCA1* and* BRCA2 *mutations.* BRCA1* carriers predominantly suffer from breast and ovarian cancers and the development of cancers for other sites is less frequent than for* BRCA2 *mutation carriers. Only few cases of pancreatic carcinomas in* BRCA1* mutation carriers were reported in the literature.* BRCA2* mutation-associated pancreatic cancer, in contrast, is described to show a lifetime risk of 0.5%. The reported relative risk to develop pancreas cancer for* BRCA1 *mutation carriers is 2.26 and 3.55 for* BRCA2 *mutation carriers [3, 7, 8]. Furthermore, there is data indicating that* BRCA2 *mutation carrying families having one case of ovarian cancer in the family are more likely to develop pancreatic and prostate cancers [7].

Primary squamous cell carcinoma of the pancreas is a rare disease with only very few cases described in the literature. As squamous cell epithelium is not present in healthy pancreas tissue, pathogenesis of this carcinoma still remains unclear and most likely reflects unscheduled differentiation of a malignant pancreatic cancer stem cell. Squamous cell carcinoma of other body parts is considered to be sensitive to radio- and chemotherapy. However, outcome in patients with primary squamous cell carcinoma of the pancreas remains basically poor [2].

As surgery remains the gold standard for management of pancreatic carcinomas in general, in our case, we firstly intended to reduce tumor burden using a neoadjuvant therapy setting. Due to their functional involvement in repair mechanisms of damaged DNA, in particular the repair of double-strand breaks,* BRCA1/2* mutation-associated tumors are thought to be sensitive to radiochemotherapy, in particular to alkylating agents [3].

Despite advanced stage disease, the tumor mass was reduced by radiochemotherapy so that the patient could undergo successful surgical resection of the mass. Seven months after neoadjuvant treatment and surgery there was no evidence of disease. Taking into account the high sensitivity to alkylating agents and the described radiation induced postoperative complications, one could consider a treatment with alkylating agents only, to watch the course of the disease and then further decide on the additional application of radiation therapy, either to add no radiation at all or to apply lower doses. Another option would be the initial usage of lower doses of radiation to prevent the described complications.

To our knowledge, this is the first case of primary squamous cell carcinoma of the pancreas in a patient with a known* BRCA2* germ line mutation. Further our case illustrates that certain subtypes of pancreatic carcinomas may occur against the background of HBOC syndrome.
